# Ab Initio Approach to the Structure, Vibrational Properties, and Electron Binding Energies of H_2_S∙∙∙SO_2_

**DOI:** 10.3390/molecules28186656

**Published:** 2023-09-16

**Authors:** Isaac O. M. Magalhães, Benedito J. C. Cabral, João B. L. Martins

**Affiliations:** 1 Computational Chemistry Laboratory, Institute of Chemistry, University of Brasilia, Brasilia 70910900, DF, Brazil; isaac.magalhaes@aluno.unb.br (I.O.M.M.); bjcabral@fc.ul.pt (B.J.C.C.); 2 Biosystems and Integrative Sciences Institute, BioISI, Faculdade de Ciências de Lisboa, 1749-016 Lisboa, Portugal

**Keywords:** vibrational spectroscopy, CCSD(T), H_2_S-SO_2_ complex

## Abstract

The present study employs high-level ab initio calculations to investigate the structure, vibrational frequencies, and electronic properties of H_2_S∙∙∙SO_2_. The analysis of vibrational frequencies reveals an intramolecular vibrational energy transfer phenomenon, where energy from the stretching modes of H_2_S is transferred to the ν1s mode of SO_2_. At the CCSD(T)/aug-cc-pVQZ level, the interaction energy between H_2_S and SO_2_ is predicted to be 2.78 kcal/mol. Electron propagator theory calculations yield a HOMO–LUMO gap of 8.24 eV for H_2_S∙∙∙SO_2_. Furthermore, by utilizing ab initio results for the adiabatic ionization energy and electron affinity, the electrophilicity of H_2_S∙∙∙SO_2_ is estimated to be 2.01 eV. This value is similar to the electrophilicity of SO_2_, suggesting comparable reactivity and chemical behavior. The non-covalent interaction (NCI) analysis of the H_2_S∙∙∙SO_2_ complex emphasizes the significant contribution of non-covalent van der Waals interactions in its energetic stabilization.

## 1. Introduction

Acid rain is a significant environmental concern that has been widely studied and discussed. It is caused by the release of sulfur dioxide (SO_2_) and other acidic pollutants into the atmosphere, which react with water vapor to form sulfuric acid (H_2_SO_4_) and other acids [1,2,3,4,5,6,7]. The natural pH of rain is close to 5.5. However, acid rain has a substantially lower pH, reaching 4.4. The low pH of this type of rain can cause structural problems in buildings and severe environmental damage, especially in primarily aquatic ecosystems [8].

Among the main sources of sulfur dioxide are industrial processes of burning fossil fuels. Sulfur organic and inorganic compounds are present in fossil fuels, with almost 3% of sulfur by weight [9]. However, sulfur supply comes from the desulfurization of fossil fuels of about 80%, which reduces the SO_2_ emission [10,11,12,13]. Volcanoes, power plants, smelters, and the oil and gas industry are the primary sources of SO_2_ [14]. H_2_S primary emission comes from organic matter in swamp areas [15,16]. Processes involving desulfurization reactions, such as the Claus reaction, are used to convert sulfur-based gases, such as H_2_S and SO_2_, into elemental sulfur. The reaction typically proceeds as follows:

$$H_{2}S+\frac{3}{2}O_{2}\overset{}{\to }{SO}_{2}+H_{2}O$$

$${2H}_{2}S+{SO}_{2}\overset{}{\to }{\frac{3}{2}S}_{2}+{2H}_{2}O$$

where the first reaction consists of the oxidation of H_2_S to SO_2_ and the second reaction is a gaseous reaction between H_2_S and SO_2_ to form the expected product. However, the second reaction can be processed to form S_2_O and H_2_O so that two new side reactions can start to occur [17]:

$$H_{2}S+S_{2}O\overset{}{\to }S_{3}+H_{2}O$$

$$H_{2}S+S_{2}O\overset{}{\to }H_{2}S_{2}O_{2}$$

It is relevant to point out the greater facility presented by thiosulfurous acid to move to the H_2_S∙∙∙SO_2_ reagents, contrary to what would be expected to move to H_2_O∙∙∙S_2_O [17].

In view of this problem, it becomes crucial to gain a comprehensive understanding of the interaction between H_2_S and SO_2_ as well as the associated energetics involved in forming the H_2_S∙∙∙SO_2_ complex. This understanding is particularly significant due to the influence of H_2_S∙∙∙SO_2_ in atmospheric chemistry and industrial contexts [17].

Applying methods developed in theoretical chemistry enables a comprehensive analysis of the structure and energetics of molecular systems, relying on a fundamental understanding of intermolecular interactions [18,19,20,21,22]. In the case of H_2_S∙∙∙SO_2_, there is evidence suggesting that the interactions occurring in this system are primarily associated with S∙∙∙S chalcogen–chalcogen interactions [23,24,25,26]. These interactions play a crucial role in determining the stability and behavior of the H_2_S∙∙∙SO_2_ complex, highlighting the significance of studying these specific intermolecular interactions at a molecular level.

Post–Hartree–Fock computational chemistry methods, such as second-order Møller–Plesset Perturbation Theory (MP2), can be employed to investigate dimers of H_2_S∙∙∙H_2_S, SO_2_∙∙∙SO_2_, and H_2_S∙∙∙SO_2_. These methods allow for determining interaction energies and the distribution of electronic density [21,27]. On the other hand, CCSD(T) (Coupled Cluster with Single and Double excitations and Triple excitations corrections) can be utilized to obtain data related to the transfer of electron density between atoms, as well as the energies involved in the process and the geometry of the system. Notably, a high degree of accuracy is observed in the calculated energies when compared with experimental data [21,23,28]. An example is the recently reported results for H_2_S∙∙∙H_2_S, where CCSD(T) results agree with the geometry and vibrational frequencies [21]. These computational methods provide valuable insights into the electronic structure, energetics, and geometries of H_2_S∙∙∙H_2_S, SO_2_∙∙∙SO_2_, and H_2_S∙∙∙SO_2_ dimers, aiding in the understanding of their properties and behavior.

Another important aspect relates to the electronic properties of H_2_S∙∙∙SO_2_, particularly the energies of the frontier orbitals. These frontier orbitals play a significant role in ionization and electron attachment processes. By studying the energies of these orbitals, valuable information can be obtained regarding the reactivity and chemical behavior of the H_2_S∙∙∙SO_2_ system. Understanding the electronic properties and the energetics of the frontier orbitals provides insights into the potential for ionization or electron attachment events, which are relevant for various chemical and environmental processes involving H_2_S∙∙∙SO_2_. In this context, electron propagator theory (EPT) is reliable for obtaining accurate orbital energies. A detailed review on the methodology and applications of EPT was provided by Ortiz [29].

The focus of the present study is to accurately determine the structure, interaction energy, and electronic properties of the H_2_S∙∙∙SO_2_ system. This aim is achieved by employing high-level ab initio methods, specifically CCSD(T) and EPT. Some emphasis was placed on the calculation of reactivity indexes such as the chemical potential, hardness, and electrophilicity, which are closely related to the ionization and electron attachment processes [30].

## 2. Results and Discussion

### 2.1. Structure, Vibrational Frequencies, and Rotational Constants

#### 2.1.1. Structure

**Figure 1:**
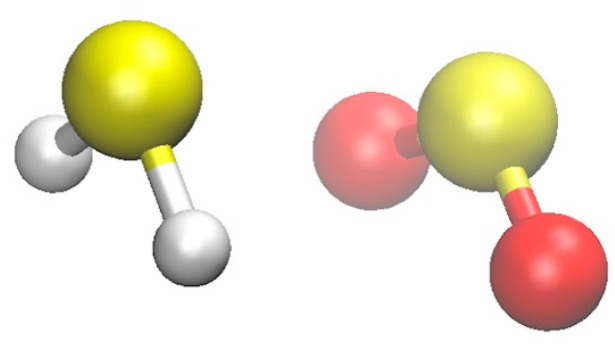
Structure of H_2_S∙∙∙SO_2_.

The structure of H_2_S∙∙∙SO_2_ is illustrated in Figure 1. The complex is stabilized by SS chalcogen–chalcogen interaction. Additional O∙∙∙H interactions should also be considered. The O∙∙∙H distances observed in H_2_S∙∙∙SO_2_ are in the range of approximately 3.1–3.3 Å, as shown in Table 1. These distances fall on the upper side of the typical hydrogen bond range, which is generally between 2.3–3.3 Å.

Intermolecular distances and angular parameters for H_2_S∙∙∙SO_2_ relying on different methods and basis sets are gathered in Table 1. Additional data for geometric parameters are reported in the Supplementary Material Tables S1–S3.

A comparison of optimized geometries at different theoretical levels and basis sets reveals that, in general, they are similar. Specifically, when using the AVQZ basis set, the predicted S∙∙∙S distance differs by less than 0.06 Å between CCSD(T) and CCSD calculations. For the same basis set, a slight increase of approximately 0.2 Å in the O∙∙∙H distance is observed when moving from MP2 to CCSD, although MP2 and CCSD(T) calculations yield similar values for this distance.

In general, a very good agreement is observed between theoretical and experimental data for the S∙∙∙S distance. The CCSD(T)/AVTZ result for the S∙∙∙S distance closely reproduces the experimental data reported by Kukolich et al. [26].

The angular parameters OS∙∙∙S and HS∙∙∙S in Table 1 show a maximum variation of 1.98 degrees for both HSH and OSO. The angles θ and φ, as defined in the work by Kukolich et al. [26], are also reported. θ represents the angle between the SO_2_ plane and the SS line, while φ represents the angle between the H_2_S plane and the SS line. Our calculated values of angle θ are smaller than the experimental values reported by Kukolich et al. [26]. One possible explanation is that their values were obtained through a fitting procedure involving rotational constants and different fittings were proposed in their work [26].

When comparing the structural parameters of the H_2_S and SO_2_ fragments with those in H_2_S∙∙∙SO_2_, it is observed that most of the changes in bond lengths and valence angles are primarily associated with the SO_2_ fragment. The SO_2_ fragment exhibits more significant changes in distances and angles compared with the relatively smaller changes observed in H_2_S.

The data obtained in MP2 calculations indicate that the variations in bond lengths for H-S bonding in the H_2_S∙∙∙SO_2_ complex compared to the monomer values are smaller than 0.2%. Similarly, the variations in the HSH angle for H_2_S in the complex are less than 0.3%. For the SO_2_ fragment, the variations in bond lengths are below 0.08% and the angle variation is within 0.6% when comparing the complex to the monomer values. Indeed, the results obtained from MP2 calculations strongly suggest that the structural changes in the H_2_S∙∙∙SO_2_ complex, when compared to the isolated H_2_S and SO_2_ molecules, are minimal. This indicates that the interaction between H_2_S and SO_2_ does not significantly alter the overall geometry of the individual molecules. These findings provide valuable insights into the nature of the H_2_S∙∙∙SO_2_ complex and its molecular behavior.

In the case of the CCSD(T) calculations, it is observed in Table 1 that the S∙∙∙S interaction distance is smaller than in the MP2 and CCSD calculations. This result suggests that the excited triple correction provides a better description of dispersion interaction. 

Table S3 shows the difference found in the H_2_S∙∙∙SO_2_ complex regarding the monomer-optimized geometry. The difference in the interatomic distances is smaller than 0.0021 Å for the distances and 3.5° for the angles. In general, the interatomic distances show the same trend for all methods. The MP2 r(SH) and r(SO) are closer to the CCSD(T) values than CCSD. Both HS and SO distances are slightly increased (almost 0.001 Å) in relation to the monomer distance, except MP2/AVDZ for the SO distance. Therefore, both bonds have weakened.

Compared with the H_2_S∙∙∙H_2_S complex, the H_2_S∙∙∙SO_2_ complex has received much less attention in studies reported in the literature, and the geometry and energies have fewer estimates. Despite the minor variations in bond distances and angles concerning the 1990 experimental data of Kukolich [26], the results of all correlation-consistent basis sets show reliability and are in better accordance than the theoretical results of the literature. Therefore, comparing with the experimental bond distance and angles, the useful geometric data obtained with CCSD(T)/aug-cc-pVTZ and CCSD(T)/aug-cc-pVQZ are suitable estimates for this complex and can be taken as a reference calculation.

#### 2.1.2. Vibrational Frequencies

Stretching frequencies of H_2_S and SO_2_ have a gap between symmetric (ν1s) and antisymmetric (ν1a) modes. Here, for both HS and SO stretching, we will define a parameter δ as ν1a-ν1s to represent the gap between the antisymmetric (ν1a) and symmetric (ν1s) stretching frequencies.

Therefore, the discussion on the vibrational frequencies will initially prioritize the δ parameter as it is a more sensitive measure for evaluating the accuracy of the calculated frequencies. We notice that theoretical frequencies are harmonic; thus, no scaling factor to consider anharmonic effects is used. Table 2 reports the difference between theoretical and experimental gaps for H_2_S and SO_2_. Vibrational frequencies (in cm^−1^) for the antisymmetric $\nu_{1a}$ and symmetric $\nu_{1s}$ stretching modes in H_2_S∙∙∙SO_2_ are reported in Supplementary Material Table S4.

For H_2_S, the deviations from experimental data are positive and relatively small, with values less than 10 cm^−1^. The results obtained from the CCSD method show better agreement with the experimental values.

On the other hand, for SO_2_, the vibrational results obtained from the MP2 method are in good agreement with the experimental data. However, the CCSD and CCSD(T) methods underestimate the gap between the symmetric and antisymmetric stretching frequencies for SO_2_. It should be observed that δ for SO_2_ presents the most significant deviations from the experimental data. Among various other sources to enhance confidence, a possible explanation for the observed differences could be the importance of including additional *d* functions in the calculation for studying structures and frequencies involving sulfur atoms [32,33]. As reported in the literature, including extra *d* functions can improve the accuracy of the estimates and better capture the electronic behavior of sulfur-containing molecules [32,33,34,35].

Despite the aforementioned importance of including extra d functions for studying structures and frequencies involving sulfur atoms, it has generally been observed that there is good agreement between theory and experiment for the structure and vibrational frequencies of H_2_S∙∙∙SO_2_. This agreement supports the accuracy of the currently employed ab initio methods in predicting the properties of these molecules. The impact of spin contamination regarding the formation of the H_2_S∙∙∙SO_2_ complex has been explored. The multireference T1 diagnostic, as defined for the CCSD wave function, is the Frobenius norm [36] and is used to establish the single-reference quality. The T1 diagnostic of CCSD/aug-cc-pVQZ is less than 0.018, which suggests that a single reference is probably enough for this system (values smaller than 0.02). As an alternative, the percentage of the molecular total atomization energy (%TAE_e_[(T)]) is of practical use to assess the nondynamic correlation and the reliability of single reference calculation [37,38]. For the aug-cc-pVQZ basis set, %TAE_e_[(T)] is less than 4.3%, which leads to confidence in using a single reference [39]. 

The strength of the interactions between H_2_S and SO_2_ in H_2_S∙∙∙SO_2_ can be related to the difference between the vibrational frequencies in the complex and monomers. Table 3 reports results for $∆\nu \equiv \nu_{H2S-SO2}-\nu_{X}$, where X = H_2_S, SO_2_.

In the case of H_2_S, both the antisymmetric stretching frequency (ν1a) and symmetric stretching frequency (ν1s) are red-shifted in the complex compared to the monomer. The CCSD method predicts smaller values for these frequencies while MP2 yields larger values. Regarding SO_2_, the antisymmetric stretching frequency (ν1a) is also red-shifted in the complex. Interestingly, the symmetric stretching frequency (ν1s) is blue-shifted, meaning it is shifted to higher frequencies compared to the monomer. The observed result suggests that an intramolecular vibrational energy transfer occurs upon complex formation [40,41]. This process involves the transfer of vibrational energy from the stretching modes of H_2_S to the ν1s mode of SO_2_. Due to this energy transfer, the antisymmetric stretching frequency of H_2_S undergoes a red-shift, while the symmetric stretching frequency of SO_2_ experiences a blue-shift. This phenomenon highlights the dynamic interplay and energy exchange between the vibrational modes of the molecules within the H_2_S∙∙∙SO_2_ complex.

### 2.2. Interaction Energies and Electronic PropertiesInteraction Energies

The complex geometry was characterized as a minimum energy structure by calculating vibrational frequencies that were real and positive. The interaction energy ${\Delta E}_{I}$ between H_2_S and SO_2_ in H_2_S∙∙∙SO_2_ was computed using this minimum geometry and is defined as

$${\Delta E}_{I}=E\left(H_{2}S\right)+S\left(SO_{2}\right)-E(H_{2}S\cdots SO_{2})$$

where the energy of the fragments E(H_2_S) and E(SO_2_) are calculated at the geometry of the H_2_S∙∙∙SO_2_ complex. Basis set superposition errors (BSSE) due to finite basis set effects were corrected using the counterpoise (CP) method, where the energies of the fragments are calculated with the same basis set of H_2_S∙∙∙SO_2_. The corrected ${\Delta E}_{I}\;$is represented as ${\Delta E}_{I}$(CP) in Table 4.

It is observed in Table 4 that the S∙∙∙S distance decreases with the increase in the basis-set size. In parallel, the analysis of energies shows a more significant increase with the basis set. Our best result for the interaction energy ${\Delta E}_{I}\left(CP\right)$ is 2.78 kcal/mol from CCSD(T)/AVQZ. Excepting an MP2/6-31G* result reported by Plummer [27] for the binding energy of H_2_S∙∙∙SO_2_ of 1.7 kcal/mol, interaction energy data are apparently not available in the literature. In the absence of precise values for the interaction energy of H_2_S∙∙∙SO_2_, it is worth noting that a DFT study [42] reported an interaction energy of 5.86 kcal/mol for the H_2_O∙∙∙SO_2_ system. This finding predicts stronger binding energy compared to the H_2_S∙∙∙SO_2_ interaction, which is the expected result.

### 2.3. Electronic Properties

#### 2.3.1. Electron Binding Energies and Reactivity Parameters

Table 5 reports the vertical and adiabatic ionization energies (IEs) and the electron affinities (EAs) of H_2_S, SO_2_, and H_2_S∙∙∙SO_2_. ∆E(CCSD) calculations were performed to account for relaxation effects during ionization or electron attachment processes, predicting adiabatic IEs and EAs. The estimates include zero-point vibrational energy (ZPVE) corrections at the MP2/AVTZ level.

Table 5 presents the results obtained using different methods: Koopman’s theorem (KT), outer valence Green’s function (OVGF), and the partial third-order (P3) approximation. The results obtained from Koopman’s theorem demonstrate the Hartree–Fock theory’s predictive power and limitations. Compared with the EPT results, it becomes evident that electronic relaxation and correlation effects, which are absent in Hartree–Fock theory, play a significant role. In general, the OVGF approximation provides results that are in better agreement with experimental data. Therefore, the discussion of orbital energies will primarily focus on the results obtained from the OVGF approximation.

The calculated ionization energy (IE) of H_2_S from the outer valence Green’s function (OVGF) method, which is 10.448 eV, is in excellent agreement with the experimental value of 10.453 ± 0.008 eV. This IE corresponds to the highest occupied molecular orbital (HOMO) energy in H_2_S. Furthermore, based on the results obtained for the electron affinities (EAs) of H_2_S, no significant electron attachment is expected to occur for this species.

Recent Electron Propagator Theory (EPT) results for SO_2_ were reported by Pawlowski and Ortiz [43]. Our calculations using the outer valence Green’s function (OVGF) method yield very good agreement with their reported values and the experimental value of 12.349 eV [44]. The vertical and adiabatic ionization energies of SO_2_ are 12.619 eV and 12.537 eV, respectively, which closely match their results of 12.614 eV and 12.427 eV. The electron affinity calculation places the LUMO energy of SO_2_ at 0.73 eV, resulting in a HOMO–LUMO (HL) gap of 11.9 eV. The adiabatic electron affinity obtained from ∆E[CCSD] calculations is 1.267 eV, which is in good agreement with the experimental value of 1.14 ± 0.05 eV reported by Rothe in 1975 [45] and 1.107 ± 0.008 eV reported by Nimlos in 1986 [46].

Regarding H_2_S-SO_2_, the OVGF results for the vertical ionization energy and electron affinity are 10.619 eV and 0.609 eV, respectively, leading to a HOMO–LUMO gap of 8.24 eV. The corresponding adiabatic values from ∆E[CCSD] calculations are 9.873 eV and 1.637 eV. Unfortunately, we are not aware of any experimental results for the ionization energy and electron affinity of H_2_S∙∙∙SO_2_.

**Table 5:** Ionization energies and electron affinities (in brackets). EPT/AVQZ calculations were carried out with CCSD/AVTZ-optimized geometries. Adiabatic values rely on CCSD/AVQZ//MP2/AVTZ calculations. Values in eV.

	Vertical			Adiabatic
	KT	OVGF	P3+	∆E [CCSD]
	IE [EA]	IE [EA]	IE [EA]	IE [EA]
H_2_S (a)	10.487 [−0.682]	10.448 [−0.507]	10.328 [−0.485]	10.343 [−0.468]
SO_2_ (b)	13.560 [−0.069]	12.619 [0.730]	12.751 [0.792]	12.537 [1.267]
H_2_S-SO_2_	10.144 [0.111]	10.129 [0.979]	9.929 [1.043]	9.873 [1.637]

(a) IE 10.453 ± 0.008 [47]. (b) VIE: 12.614 OVGF AIE 12.437 (D-CCSD) [43]. (b) AEA: 1.14±0.05 [45].

**Figure 2:**
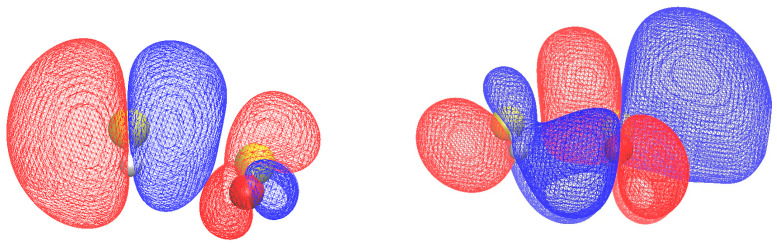
HOMO (**left**) and LUMO (**right**) of H_2_S-SO_2_. The isodensity is 0.005 ($e/a_{0}^{3}$).

Figure 2 depicts a representation of the HOMO and LUMO of H_2_S-SO_2_. The HOMO exhibits a localized S p-orbital primarily confined to the H_2_S fragment. On the other hand, the complex structure of the LUMO demonstrates spatial delocalization and p-d orbital mixing, primarily involving the SO_2_ moiety.

A fundamental property for evaluating the affinity for electrons of a given species is its electrophilicity. However, unlike electron affinity (EA), which is concerned with the attachment of a single electron, electrophilicity assesses the energetic stabilization of a ligand in relation to the electron flow between donor and acceptor species [30].

The electrophilicity (ω) can be defined [30] as the relation between chemical potential and the global hardness:

$${\omega =\mu }^{2}/2\eta$$

where the chemical potential μ and the chemical hardness η are given by

μ=−(IE+EA)/2    and  η=(IE−EA)/2

Table 6 reports the results for μ,η and ω, which are calculated using the CCSD/AVQZ//MP2/AVTZ values of ionization energies and electron affinities reported in Table 5. These reactivity descriptors are valuable in studying complex formation, where the maximum hardness principle correlates the largest hardness with the stability of a system [48,49]. 

For SO_2_ electrophilicity, the deviation from experimental results is less than 0.2 eV, indicating good agreement. Additionally, the electrophilicity value for H_2_S∙∙∙SO_2_ (4.227 eV) is quite similar to the one predicted for SO_2_, suggesting that the electron flow from the environment to H_2_S∙∙∙SO_2_ is mainly determined by the SO_2_ fragment. Fukui functions are depicted in Figure S1 and show the same trend of electrophilicity. 

#### 2.3.2. Electrostatic Potential and Non-Covalent Interactions (NCI) Analysis

**Figure 3:**
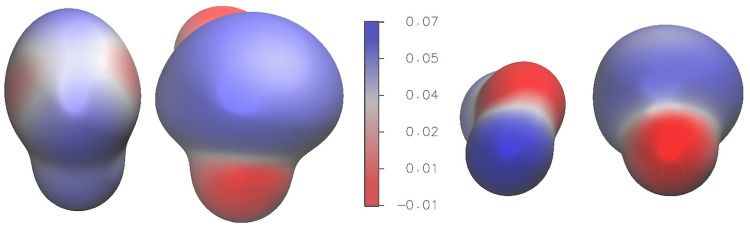
Electrostatic potential over isodensity surfaces. Left: H_2_S∙∙∙SO_2_; right: H_2_O∙∙∙SO_2_. The isodensity is 0.004 e/ao^3^. Electrostatic potential in a.u.

Figure 3 presents the electrostatic potential (ESP) plotted on an isodensity surface for the H_2_S∙∙∙SO_2_ complex (left panel) and the H_2_O∙∙∙SO_2_ complex (right panel). In the case of H_2_S∙∙∙SO_2_, the polarization effects arising from the difference between positive and negative regions of the electrostatic potential are primarily localized on the SO_2_ monomer. This indicates that the interactions between H_2_S and SO_2_ predominantly involve the polarization of the SO_2_ molecule. In contrast, for the H_2_O∙∙∙SO_2_ complex, significant polarization effects are observed on both the H_2_O and SO_2_ monomers. 

**Figure 4:**
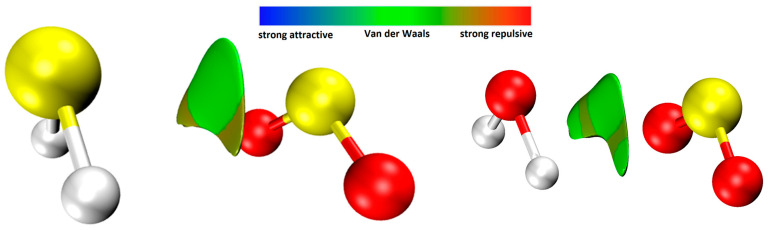
NCI analysis of the H_2_S∙∙∙SO_2_ interactions. Left: H_2_S∙∙∙SO_2_; right: H_2_O∙∙∙SO_2_. Color labels: yellow sulfur, red oxygen, and white hydrogen.

One of the analyses utilized to characterize the nature of the interaction between two molecules is non-covalent interactions (NCI) analysis [50], as described by Contreras-García et al. [51]. This analysis examines the interaction on the same surface and provides valuable insights into the non-covalent forces at play. The results of this analysis are presented in Figure 4, which showcases the specific characteristics and distribution of the non-covalent interactions between H_2_S and SO_2_ within the complex.

The NCI analysis depicted in Figure 4 highlights the significance of weak van der Waals interactions in energetically stabilizing the H_2_S∙∙∙SO_2_ complex. These interactions play a crucial role in the overall stability and binding of the complex. Additionally, the analysis reveals a depletion of electronic density between the fragments, indicating a characteristic feature of non-covalent interactions. It is important to note that there is also a presence of strong repulsion in certain regions of the same surface, as indicated by the brown region in Figure 4. This suggests the existence of repulsive forces between specific parts of the H_2_S and SO_2_ molecules within the complex. The interplay between attractive van der Waals interactions and repulsive forces contributes to the overall energetics and structural characteristics of the H_2_S∙∙∙SO_2_ complex. Interestingly, NCI analysis for the H_2_O∙∙∙SO_2_ complex also shows that non-covalent interactions play a significant role in the energetic stabilization of this system. This suggests that the natures of the intermolecular SS and SO interactions exhibit some similarity.

## 3. Materials and Methods

The structures of H_2_S, SO_2_, and H_2_S∙∙∙SO_2_ were optimized using three different methods: MP2 (Møller–Plesset second-order perturbation theory) [52,53], CCSD (coupled cluster with single and double excitations) [54,55], and CCSD(T) (coupled cluster with single and double excitations, and correction to triple excitations) [56].

The MP2 and CCSD calculations were performed using the Gaussian16 program package [57]. On the other hand, the CCSD(T) calculation was carried out using the CFOUR program with analytical second derivatives [58].

To perform these calculations, three different levels of basis sets were used: aug-cc-pVTZ (AVTZ), aug-cc-pVQZ (AVQZ), and aug-cc-pV5Z (AV5Z). These basis sets are known as the Dunning correlation-consistent basis sets [59], and they provide increasingly accurate results including more basis functions from double-zeta to quintuple-zeta.

The use of monoelectronic basis sets in these calculations can lead to an overestimation of interaction energies due to the finite size of the basis sets and the different variational spaces of the complex (H_2_S∙∙∙SO_2_) and fragments (H_2_S and SO_2_). To address this issue, it is necessary to apply the Counterpoise (CP) correction method, which helps estimate the influence of the Basis Set Superposition Error (BSSE) [60]. The nature of the interaction between the H_2_S and SO_2_ monomers in H_2_S∙∙∙SO_2_ was discussed by non-covalent interactions (NCI) analysis [51].

Electron propagator theory (EPT) calculations were performed using the outer valence Green’s function (OVGF) and partial third-order (P3) approximations [29]. EPT is known to provide accurate estimates of orbital energies. EPT calculations were carried out with the Gaussian16 program [28].

The adiabatic ionization energies and electron affinities were predicted through ∆E calculations, where the energy difference between the neutral and charged species was calculated. Optimized geometries for the neutral, cationic, and ionic species were determined at the MP2/AVTZ level, and frequencies were calculated at the same level. Finally, energies were estimated at the CCSD/AVQZ//MP2/AVTZ level. Chemical reactivity indexes [30] were calculated using ionization energies and electron affinities from ∆E(CCSD) calculations.

## 4. Conclusions

The present study employs high-level ab initio calculations to investigate the structure, vibrational frequencies, and electronic properties of the H_2_S∙∙∙SO_2_ complex. The analysis of vibrational frequencies reveals an intramolecular vibrational energy transfer phenomenon, where energy from the stretching modes of H_2_S is transferred to the ν1s mode of SO_2_. At the CCSD(T)/aug-cc-AVQZ level, the interaction energy between H_2_S and SO_2_ is predicted to be 2.78 kcal/mol. This provides insight into the strength of the interaction and the stability of the complex.

Electron propagator theory calculations yield a HOMO–LUMO gap of 8.24 eV for H_2_S∙∙∙SO_2_. This information sheds light on the electronic properties and the energy required for ionization and electron attachment processes. Furthermore, by utilizing ab initio results for the adiabatic ionization energy and electron affinity, the electrophilicity of H_2_S-SO_2_ is estimated to be 2.01 eV. This value is found to be similar to the electrophilicity of SO_2_, suggesting comparable reactivity and chemical behavior.

Overall, the combination of ab initio calculations and analysis provides valuable insights into the structure, vibrational dynamics, and electronic properties of the H_2_S∙∙∙SO_2_ complex, contributing to a deeper understanding of this system. The non-covalent interactions (NCI) analysis conducted on the H_2_S∙∙∙SO_2_ complex emphasizes the significant contribution of non-covalent van der Waals interactions in its energetic stabilization.

## Figures and Tables

**Table 1:** Structure of H_2_S∙∙∙SO_2_: intermolecular distances (Å) and angles (degrees).

	S∙∙∙S	H∙∙∙O	OS∙∙∙S	HS∙∙∙S	θ	ϕ
MP2/AVTZ	3.414	3.107	89.27	76.78	88.623	70.768
MP2/AVQZ	3.387	3.137	90.11	78.40	90.215	73.140
MP2/AV5Z	3.382	3.156	90.33	79.24	90.615	74.288
CCSD/AVTZ	3.499	3.315	90.48	81.83	90.619	77.391
CCSD/AVQZ	3.481	3.335	90.70	82.37	91.324	78.983
CCSDT/AVTZ	3.454	3.181	89.25	78.43	88.558	73.168
CCSDT/AVQZ	3.427	3.221	90.12	80.33	90.248	75.960
Other Values						
Matsumura (a)	3.520	3.145			99.0(13)	56.8(11)
Kukolich (b)	3.45(1)				103(1)	71(3)
Ford (c)	3.802		72.88	67.28		
Plumer (d)	3.577	3.07				

(a) Experimental [31]; (b) Experimental [26]; (c) MP2/6-311G++(d,p) [23]; (d) MP2/6-31G* [27].

**Table 2:** Difference between theoretical and experimental data for the antisymmetric–symmetric gap δ (in cm^−1^) for the H_2_S and SO_2_ monomers.

	AVTZ	AVQZ	AV5Z
H_2_S	$\delta_{th}-\delta_{expt}$	$\delta_{th}-\delta_{expt}$	$\delta_{th}-\delta_{expt}$
MP2	8.92	8.57	8.21
CCSD	4.19	3.56	
CCSD(T)	5.40	5.07	
SO_2_	$\delta_{th}-\delta_{expt}$	$\delta_{th}-\delta_{expt}$	$\delta_{th}-\delta_{expt}$
MP2	−4.59	2.39	8.15
CCSD	−17.96	−10.98	
CCSD(T)	−15.13	−7.40	

**Table 3:** Difference ∆ν for the antisymmetric and symmetric stretching frequencies between H_2_S∙∙∙SO_2_ and monomers.

	MP2			CCSD		CCSD (T)	
	AVTZ	AVQZ	AV5Z	AVTZ	AVQZ	AVTZ	AVQZ
HS							
${∆\nu }_{1a}$	−14.60	−16.05	−11.76	−3.74	−5.86	−7.06	−6.71
${∆\nu }_{1s}$	−13.88	−15.43	−11.29	−3.31	−5.93	−6.79	−6.62
SO							
${∆\nu }_{1a}$	−1.60	−3.02	−3.76	−6.12	−5.83	−5.50	−5.86
${∆\nu }_{1s}$	5.80	5.20	4.47	1.34	1.68	1.34	1.68

**Table 4:** Interaction energies (kcal/mol) and S∙∙∙S distances (Å) for the complexes.

	S∙∙∙S	${\Delta E}_{I}$	BSSE	${\Delta E}_{I}(CP)$
MP2/AVTZ	3.4142	3.2268	0.4757	2.7600
MP2/AVQZ	3.3866	3.1661	0.2333	2.9500
MP2/AV5Z	3.3822	3.1021	0.1205	3.0000
CCSD/AVTZ	3.4993	2.7071	0.3788	2.3400
CCSD/AVQZ	3.4805	2.6006	0.1558	2.4600
CCSD(T)/AVTZ	3.4543	3.0396	0.4473	2.6000
CCSD(T)/AVQZ	3.4275	2.9537	0.1831	2.7800

**Table 6:** Chemical potential (μ), hardness (η), and electrophilicity (ω). Values in eV. Experimental values in brackets.

	$\mu =-\frac{IE+EA}{2}$	$\eta =\frac{IE-EA}{2}$	ω=μ22η
H_2_S	−4.937	5.406	2.255
SO_2_	−6.902	5.635	4.227 [4.027]
H_2_S-SO_2_	−5.755	4.118	4.021

## Data Availability

Data are available upon request.

## Supplementary Materials

The following supporting information can be downloaded at: https://www.mdpi.com/article/10.3390/molecules28186656/s1, Figure S1: Fukui functions plotted for the complex using CCSD(T)/aug-cc-pVQZ; Table S1: Interatomic distances (Å) and bond angles (degrees) the H_2_S∙∙∙SO_2_ complex; Table S2: Angles (degrees) in SO_2_, H_2_S and in the SO_2_∙∙∙H_2_S complex, where θ and φ are defined as in Ref [10]; Table S3 Difference of bond distances and angles of H_2_S and SO_2_ in relation to the monomer; Table S4: Vibrational frequencies (in cm^−1^) for the antisymmetric *v*_1a_ and symmetric *v*_1s_ stretching modes in H_2_S∙∙∙SO_2_.
