# Supplementary material for: Ab Initio Approach to the Structure, Vibrational Properties, and Electron Binding Energies of H2S∙∙∙SO2
Source: Molecules. 2023 Sep 16;28(18):6656. doi: 10.3390/molecules28186656 (PMC10535889; doi:10.3390/molecules28186656)
Supplement: Supplementary file 1 [file molecules-28-06656-s001.zip › molecules-2596838-supplementary.pdf]

molecules

Article

Ab initio approach to the structure, vibrational properties, and electron binding energies of $H_2S\cdots SO_2$.

Isaac O. M. Magalhães [1], B. J. C. Cabral [2] and João B. L. Martins [3,*]

[1] Computational Chemistry Laboratory, Institute of Chemistry, University of Brasilia, Brasilia, DF, 70910900, Brazil isaac.magalhaes@aluno.unb.br

[2] Biosystems and Integrative Sciences Institute, BioISI, Faculdade de Ciências de Lisboa, 1749-016 Lisboa, Portugal and Institute of Chemistry, University of Brasilia, Brasilia, DF, Brazil; bjcabral@fc.ul.pt

[3] Computational Chemistry Laboratory, Institute of Chemistry, University of Brasilia, Brasilia, DF, 70910900, Brazil, e-mail: lopes@unb.br

*Correspondence: e-mail@e-mail.com; Tel.: (optional; include country code; if there are multiple corresponding authors, add author initials)

Citation: To be added by editorial staff during production.

Academic Editor: Firstname Lastname

Received: date
Revised: date
Accepted: date
Published: date

Copyright: © 2023 by the authors. Submitted for possible open access publication under the terms and conditions of the Creative Commons Attribution (CC BY) license (https://creativecommons.org/licenses/by/4.0/).

Supplementary Materials:

Table S1 – Interatomic distances (Å) and bond angles (degrees) the $H_2S\cdots SO_2$ complex.

	r(SO)	r(SH)	H···O	r(S···S)	r(A···B)
MP2/AVTZ	1.4643	1.3377	3.8076	3.4143	3.4006
MP2/AVQZ	1.4522	1.3357	3.1373	3.3866	3.3850
MP2/AV5Z	1.4447	1.3343	3.1563	3.3822	3.3838
CCSD/AVTZ	1.4425	1.3404	3.3147	3.4993	3.5037
CCSD/AVQZ	1.4306	1.3382	3.3350	3.4805	3.4905
CCSDT/AVTZ	1.4562	1.343	3.1813	3.4543	3.4420
CCSDT/AVQZ	1.4437	1.3409	3.2210	3.4274	3.4283
Exp.[11]				3.45	
MP2/6-311++G(d,p) [8]				3.802	
MP2/6-31G*[12]	1.4767/1.4769	1.3389/1.3393	3.07	3.5768	
Exp[16]			3.145	3.520	3.534

(A···B) stands for the distance regarding the center of mass

Table S2. Angles (degrees) in SO_2, H_2S and in the $SO_2\cdots H_2S$ complex, where θ and ϕ are defined as in Ref. [10].

	OSO	HSH	OS···S	S···SH	θ	ϕ
MP2/AVTZ	118.19	92.23	89.27	76.77	88.623	70.768
MP2/AVQZ	118.60	92.30	90.11	78.40	90.215	73.140
MP2/AV5Z	119.00	92.31	90.33	79.24	90.615	74.288
CCSD/AVTZ	117.89	92.61	90.48	81.83	90.619	77.391
CCSD/AVQZ	118.32	92.72	90.70	82.37	91.324	78.983
CCSDT/AVTZ	117.84	92.33	89.25	78.43	88.558	73.168
CCSDT/AVQZ	118.27	92.45	90.12	80.33	90.248	75.960
Other values						
Expt.					103(a); 99.0(b)	71(a); 56.8(b)

MP2/6311++G(d,p) (c)	118.6	92.0	72.9	67.3
MP2/6-31G (d)	119.3	94.0		

(a) [11]; (b) [16]; (c) [8] (d) [12]

Table S3 Difference of bond distances and angles of H_2S and SO_2 in relation to the monomer.

Delta	DSO	DSH	DOSO	DHSH
MP2/AVTZ	0.0007	0.0019	0.004	0.152
MP2/AVQZ	0.0009	0.0021	-0.227	-0.676
MP2/AV5Z	0.0010	0.0015	0.062	-0.631
CCSD/AVTZ	0.0009	0.0006	0.018	-0.500
CCSD/AVQZ	0.0008	0.0010	0.119	-0.499
CCSDT/AVTZ	0.0009	0.0011	0.037	-0.537
CCSDT/AVQZ	0.0010	0.0012	0.079	-0.549

DSO=r(SO)dimer-r(SO)monomer; DSH= r(SH)dimer-r(SH)monomer; DOSO= r(SO)dimer-r(SO)monomer; DHSH= r(SH)dimer-r(SH) monomer;

Table S4. *Vibrational frequencies (in cm^{-1}) for the antisymmetric ν_{1a} and symmetric ν_{1s} stretching modes in $H_2S\cdots SO_2$.*

	H_2S		SO_2	
	$\nu 1a$	$\nu 1s$	$\nu 1a$	$\nu 1s$
MP2/AVTZ	2781.4	2762.2	1303.9	1104.9
MP2/AVQZ	2783.0	2764.0	1327.9	1122.7
MP2/AV5Z	2787.0	2768.3	1346.9	1136.0
CCSD/AVTZ	2742.8	2728.1	1392.6	1207.0
CCSD/AVQZ	2748.1	2733.5	1419.0	1226.5
CCSDT/AVTZ	2720.3	2704.2	1326.6	1138.6
CCSDT/AVQZ	2725.5	2709.5	1355.5	1160.1

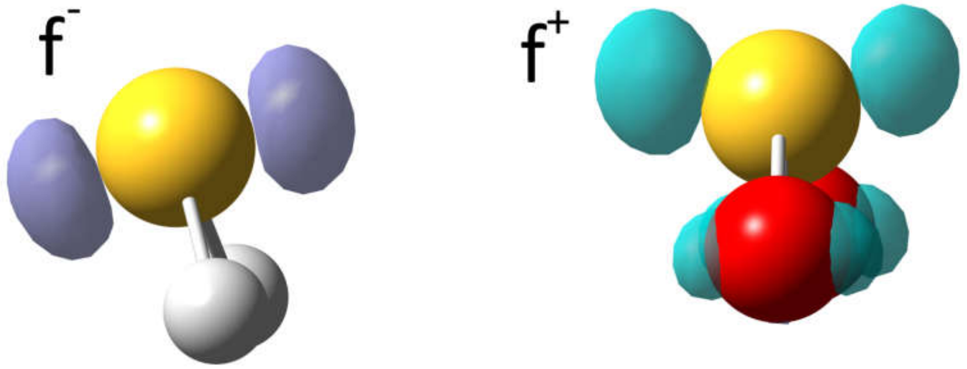

Figure S1 – Fukui functions plotted for the complex using CCSD(T)/aug-cc-pVQZ.
